# Identification of Drug Targets and Agents Associated with Ferroptosis-related Osteoporosis through Integrated Network Pharmacology and Molecular Docking Technology

**DOI:** 10.2174/0113816128288225240318045050

**Published:** 2024-03-20

**Authors:** Kailun Huo, Yiqian Yang, Tieyi Yang, Weiwei Zhang, Jin Shao

**Affiliations:** 1 Postgraduate Training Base in Shanghai Gongli Hospital, Ningxia Medical University, Yinchuan, Ningxia Hui-Autonomous Region 750004, China;; 2 Department of Orthopedics, Shanghai Pudong New Area Gongli Hospital, Shanghai 200135, China;; 3 School of Gongli Hospital Medical Technology, University of Shanghai for Science and Technology, Shanghai 200093, China;; 4 Department of Urology, Ren Ji Hospital, Shanghai Jiao Tong University School of Medicine, Shanghai 200127, China

**Keywords:** Osteoporosis, ferroptosis, resveratrol, bioinformatics, network pharmacology, molecular docking

## Abstract

**Background:**

Osteoporosis is a systemic bone disease characterized by progressive reduction of bone mineral density and degradation of trabecular bone microstructure. Iron metabolism plays an important role in bone; its imbalance leads to abnormal lipid oxidation in cells, hence ferroptosis. In osteoporosis, however, the exact mechanism of ferroptosis has not been fully elucidated.

**Objective:**

The main objective of this project was to identify potential drug target proteins and agents for the treatment of ferroptosis-related osteoporosis.

**Methods:**

In the current study, we investigated the differences in gene expression of bone marrow mesenchymal stem cells between osteoporosis patients and normal individuals using bioinformatics methods to obtain ferroptosis-related genes. We could predict their protein structure based on the artificial intelligence database of AlphaFold, and their target drugs and binding sites with the network pharmacology and molecular docking technology.

**Results:**

We identified five genes that were highly associated with osteoporosis, such as TP53, EGFR, TGFB1, SOX2 and MAPK14, which, we believe, can be taken as the potential markers and targets for the diagnosis and treatment of osteoporosis. Furthermore, we observed that these five genes were highly targeted by resveratrol to exert a therapeutic effect on ferroptosis-related osteoporosis.

**Conclusion:**

We examined the relationship between ferroptosis and osteoporosis based on bioinformatics and network pharmacology, presenting a promising direction to the pursuit of the exact molecular mechanism of osteoporosis so that a new target can be discovered for the treatment of osteoporosis.

## 1 INTRODUCTION

Osteoporosis, a chronic systemic bone disease, is characterized by loss of bone mass and destruction of bone microstructure, resulting in decreased bone mineral density (BMD) and, ultimately, an increased risk of fragility fractures [1]. The fact is that BMD declines with age; one in three women and one in five men aged over 50, respectively, are likely to experience an osteoporosis-related fracture. The hip is the most common site for osteoporosis fractures, which can seriously affect quality of life and even death [2]. The skeletal system is not static, maintaining a dynamically balanced organ until the balance is broken, causing a gradual loss of bone mass. The problem, however, is that osteoporosis produces few symptoms and is not noticed in many cases until the occurrence of a fracture. The common risk factors of osteoporosis refer to age, heredity, malnutrition, obesity and various underlying diseases [3]. The pathogenesis of osteoporosis involves a variety of biological functions, among which is ferroptosis [4, 5].

The past decade has witnessed a rapid increase in the incidence of ferroptosis, a unique mode of cell death. The imbalance of cellular metabolism and redox homeostasis drives lipid peroxidation, which ultimately leads to ferroptosis [6]. The abundance of phospholipid substrates, which drive and eliminate phospholipid peroxidation, plays a key role in regulating ferroptosis [7]. Ferroptosis has been reported to be implicated in a variety of diseases, including cancer [8], inflammation [9], cardiovascular disease [10], as well as osteoporosis [11], in which the osteoclasts could be stimulated by RANKL under normal oxygen concentration to increase the expression of such ferroptosis-related genes as Tfr1, SLC7A11 and GPX4 [11]. In the process of ferroptosis, the Wnt signaling pathway, which induces osteoblast differentiation and inhibits osteoclast proliferation, has been found to play a role in the downstream of ferroptosis to prevent intracellular iron overload, inhibit the generation of reactive oxygen species (ROS), prevent lipid peroxidation, and protect osteoblast differentiation [12].

In the current study, we analyzed two relevant datasets from the Gene Expression Omnibus (GEO) database, in which differentially expressed genes were screened using GSE35958. Consequently, protein-protein interaction (PPI) network was constructed so that Hub genes were screened out. From the ferroptosis-related database, the ferroptosis Hub genes were screened out, which were analyzed based on Gene Ontology (GO) and Kyoto Encyclopedia of Genes and Genomes (KEGG), respectively. On the basis of the dataset GSE56815, subsequently, the genes were analyzed for expression verification and diagnostic value.

**Fig. (1):**
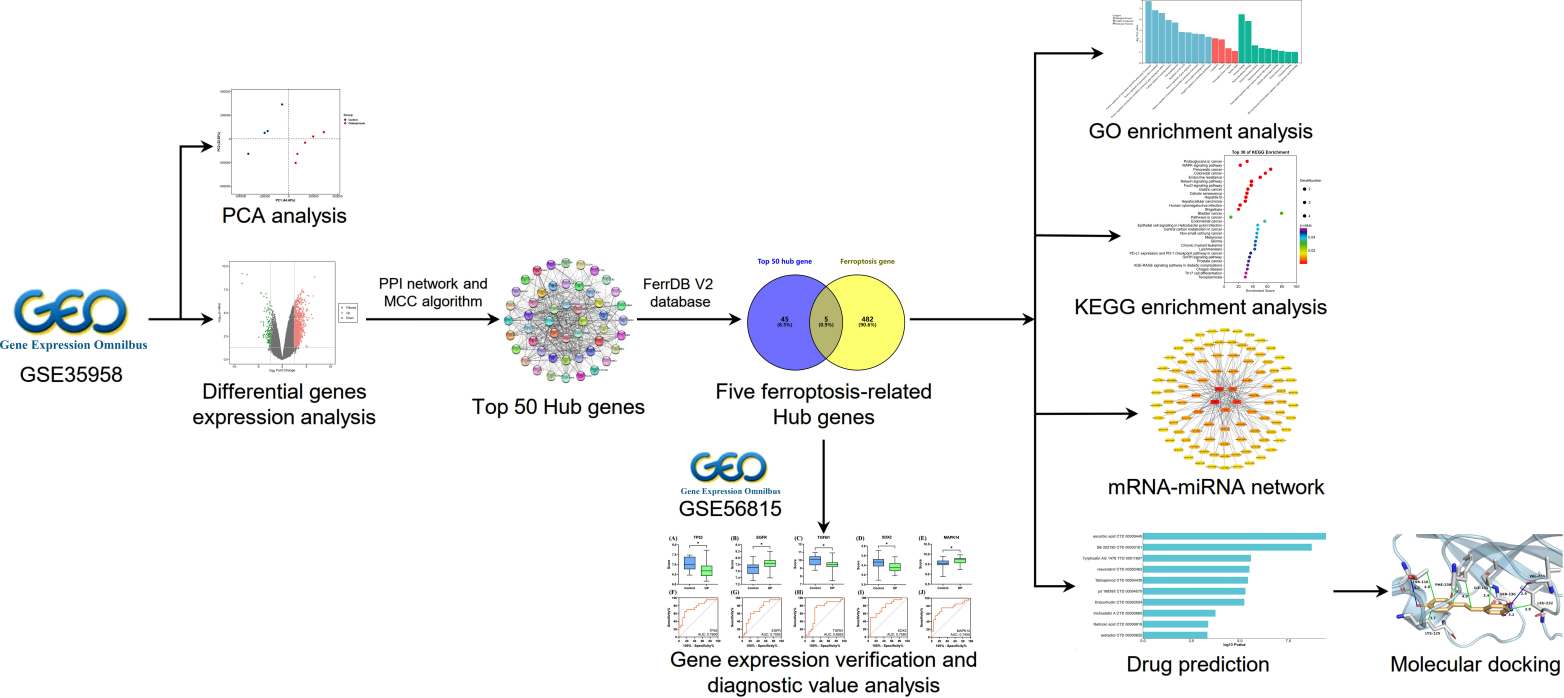
Flow diagram of the research design.

The mRNA-miRNA network was constructed to predict the potential regulatory effects of miRNAs on ferroptosis Hub genes and indicate their connections. Based on DSigDB database, the promising drugs were explored, which could target the ferroptosis Hub genes. After that, the molecular docking technology was used to explore the potential therapeutic effect of resveratrol in ferroptosis-related osteoporosis; therefore, the promising potential of treating osteoporosis can be explored by targeting ferroptosis (Fig. **1**).

## 2 MATERIALS AND METHODS

### 2.1 Data Collection

The osteoporosis-related datasets GSE35958 and GSE56815 were obtained from the GEO database [13], GSE35958 being a human bone marrow mesenchymal stem cell dataset consisting of five elderly patients with osteoporosis and four elderly volunteers with normal BMD. The platform was GPL570 (HG-U133_Plus_2) Affymetrix Human Genome U133 Plus 2.0 Array. The candidate Hub genes were validated using the GSE56815 dataset, which was composed of 20 elderly osteoporosis patients and 20 controls with normal BMD. The platform was GPL96 (HG-U133A) Affymetrix Human Genome U133A Array. From FerrDb database were identified a total of 487 ferroptosis-related genes [14].

### 2.2 Different Gene Expression Analysis

GEO2R (https://www.ncbi.nlm.nih.gov/geo/geo2r/) was used to process the GSE35958 dataset, from which the samples were divided into the osteoporosis group and normal control group for preliminary analysis to obtain the differential expression genes. Box charts, heat maps, and volcano maps were drawn using the heatmap and ggplot2 packages in the R software (Version 3.6.3). Selection criteria: |log2FC| > 2.5 and *p* < 0.05.

### 2.3 PPI Analysis of Differential Genes and Screening of Ferroptosis-related Genes

The STRING database was applied to the analysis of all differentially expressed genes that met the screening criteria [15]. In Cytoscape software (Version 3.9.1) [16], the cytohubba plug-in and MCC algorithm were performed to screen out 50 most connected Hub genes for the construction of the PPI network. The 50 Hub genes were compared with ferroptosis-related genes, with Venn diagram drawn *via* Venny 2.1.

### 2.4 GO and KEGG Pathway Enrichment Analysis

DAVID database was applied to the performance of GO and KEGG pathway enrichment analysis of the ferroptosis-related Hub genes [17], with the bubble plots and bar graphs drawn on the ggplot2 package of R software.

### 2.5 Validation of Ferroptosis Hub Genes

On GraphPad Prism 9 software verified the expression and diagnostic value of the ferroptosis Hub gene in GSE56815 dataset. The Boxplots were drawn to compare the expression levels of ferroptosis Hub genes between the osteoporosis and normal samples, with the receiver operating characteristic curve drawn and quantified by the area under the ROC curve (AUC).

### 2.6 Construction of mRNA-miRNA Regulatory Network

To predict miRNAs regulating ferroptosis Hub genes, miRNet database was used. On Cytoscape software [18], mRNA-miRNA regulatory network maps were produced.

### 2.7 Drug Prediction and Molecular Docking

DSigDB database was used to predict the potential targeted drugs for the ferroptosis-related Hub genes [19]. We ranked the predicted compounds according to their combined scores and showed the top 10 compounds in a bar chart. The molecular structure of resveratrol was obtained from the PubChem database [20], and the protein structures of ferroptosis-related Hub genes from the AlphaFold Protein Structure Database [21]. AutoDockTools was used to preprocess the protein receptors and set the molecular docking parameters; AutoDock Vina [22, 23], to perform the molecular docking and construct the model with the lowest scoring in the docking; and Pymol, to visualize the results of the molecular docking.

### 2.8 Statistical Analyses

The statistical analyses were performed using R Software (version 3.6.3) and Graph Pad Prism 9 (GraphPad Software, USA), with *p* < 0.05 considered to indicate a statistically significant difference.

## 3 RESULTS

### 3.1 Overview and Difference Analysis of Data Sets

**Fig. (2):**
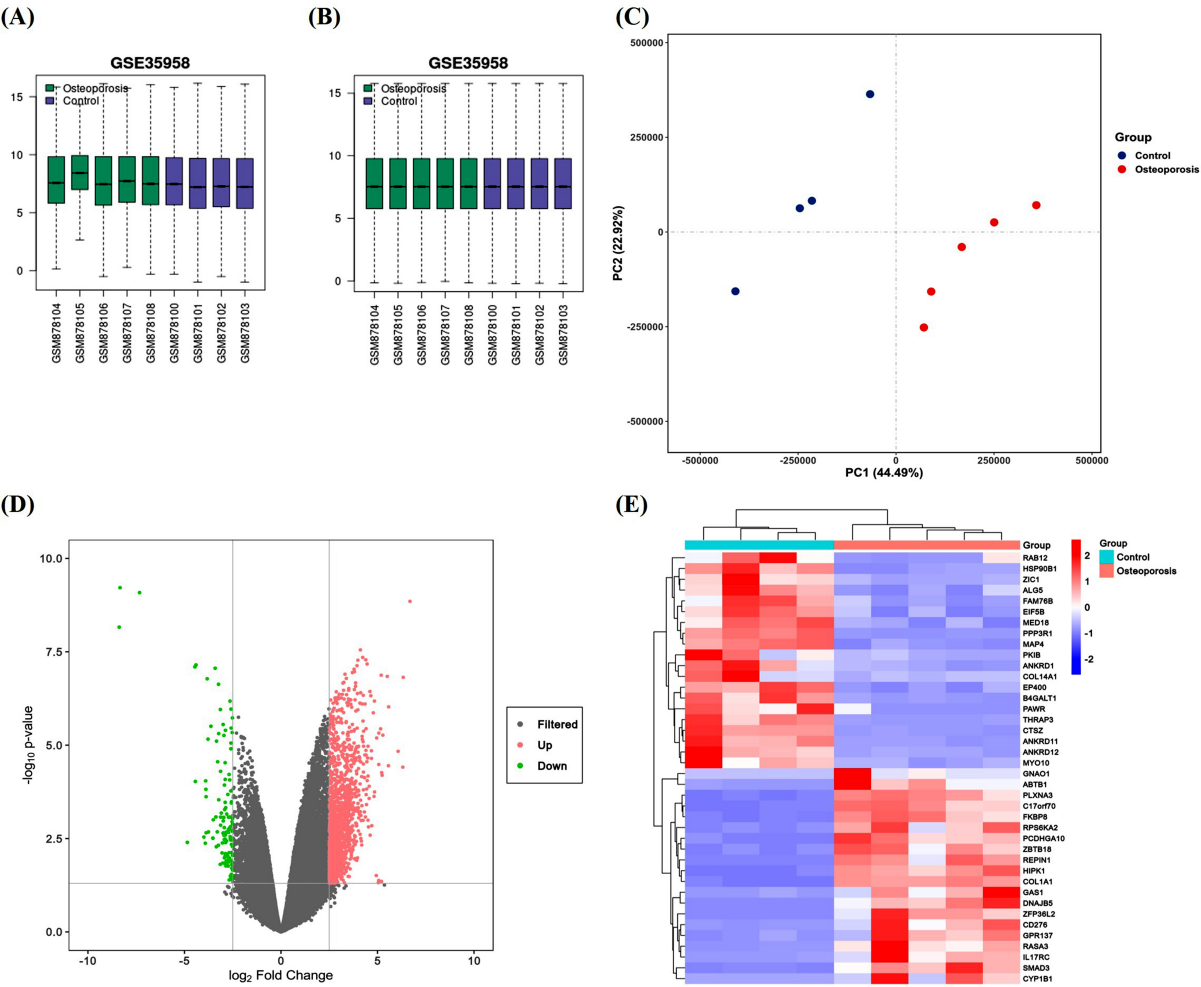
The diagram of dataset overview and difference analysis. (**A**) Unnormalized boxplot, (**B**) Normalized boxplot, (**C**) The diagram of two-dimensional principal component analysis, (**D**) Volcano plot of the expression profile of GSE35958 dataset (selection criteria: |log2FC| > 2.5, *p* < 0.05), and (**E**) The heat map of the expression profile of GSE35958 dataset.

In this study, all samples in the GSE35958 dataset were divided into osteoporosis group and normal control group, with 4 and 5 cases, respectively. After the standardized processing and box-plot drawing, the distribution of each sample was basically straight, indicating that the normalization degree was satisfactory between the samples (Figs. **2A**, **B**). In the two-dimensional principal component analysis, it was found that the separation was far apart between the two groups, indicating that the differences were significant between the groups. However, the samples were close to each other within the same group, which indicated that they were relatively similar in nature (Fig. **2C**).

We adopted two screening criteria of genetic variation: |log2FC| > 2.5, *p* < 0.05, detecting a total of 1,361 genes of significant difference (Fig. **2D**), of which each top 20 that produced high and low expression were selected to make a heat map for comparison (Fig. **2E**).

### 3.2 PPI Network Analysis and Ferroptosis Hub Gene Screening

**Fig. (3):**
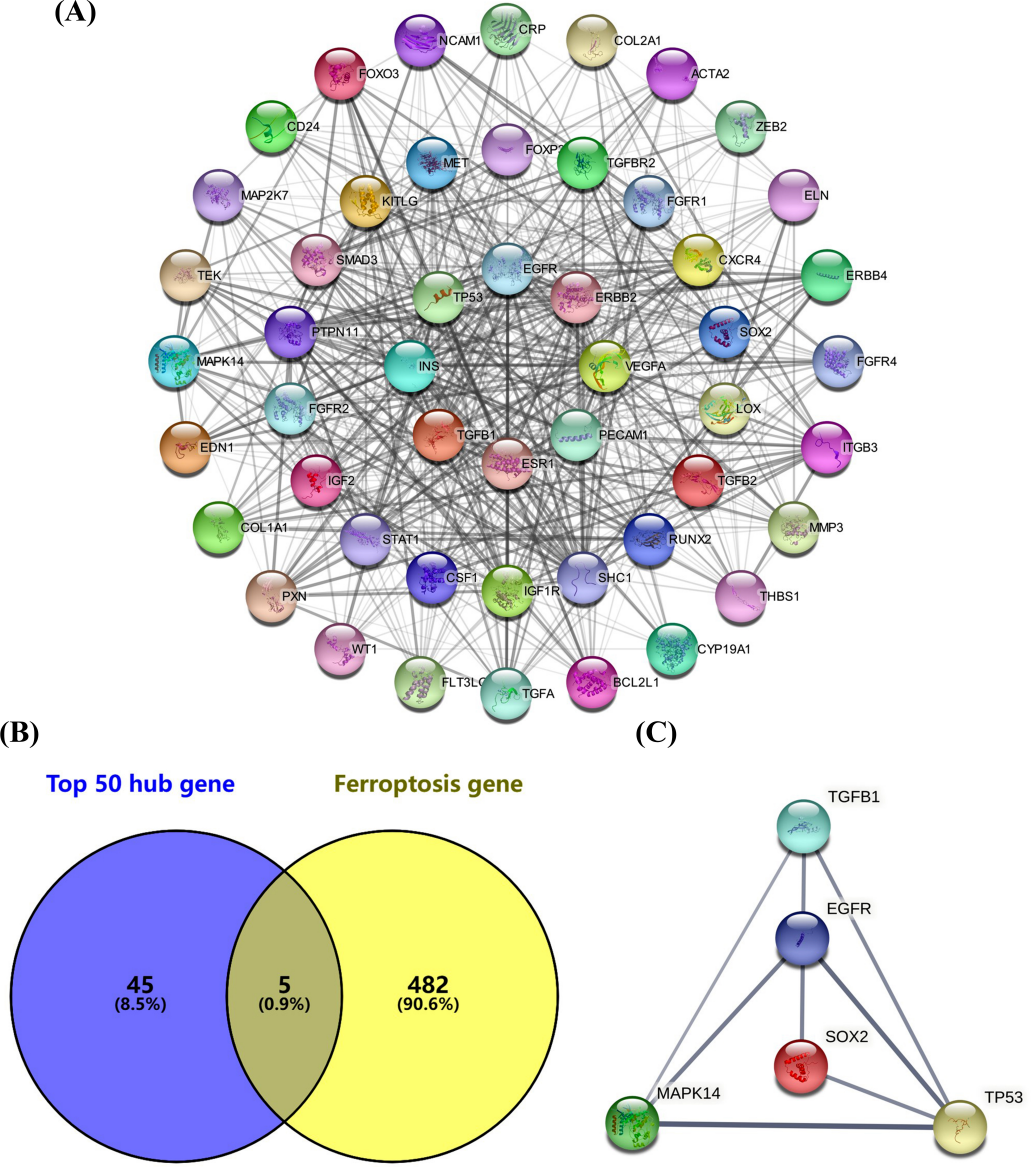
PPI network diagram of differential genes and Venn diagram of ferroptosis-related Hub genes. (**A**) PPI network of the top 50 Hub genes, (**B**) Venn diagram of the top 50 Hub genes compared with the ferroptosis-related genes, and (**C**) The diagram of PPI network of the five ferroptosis-related Hub genes.

An analysis was made of the 1,361 differentially expressed genes based on STRING database, the results showing that there were 1,081 nodes and 4,487 interactions, respectively. Then, the data were imported into cytoHubba plug-in in cytoscape (v3.9.1) software, with the MCC algorithm, applied to the marking of the top 50 genes as Hub genes, and the PPI network drawn according to the degree of connection (Fig. **3A**). After that, the ferroptosis-related genes were taken to match the top 50 Hub genes, the results of which indicated that there were five genes overlapped: TP53, EGFR, TGFB1, SOX2 and MAPK14 (Fig. **3B**). To show the interaction of these five genes, PPI network diagram was drawn (Fig. **3C**). At last, TP53, EGFR, TGFB1, SOX2 and MAPK14 were selected as Hub genes which were associated with ferroptosis in osteoporosis.

### 3.3 GO and KEGG Pathway Enrichment Analysis

**Fig. (4):**
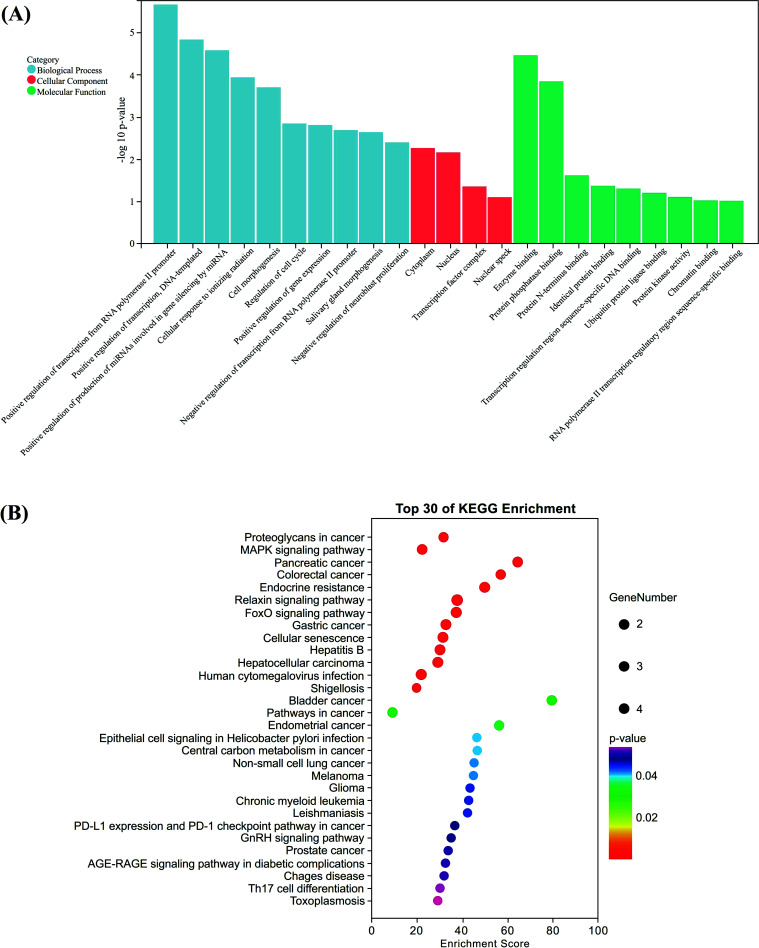
The map of GO and KEGG pathway enrichment analysis. (**A**) The graph of GO enrichment analysis: biological process (blue), cell composition (red) and molecular function (green), (**B**) The map of the top 30 from KEGG enrichment analysis.

For these five genes, we performed GO and KEGG enrichment analyses. GO enrichment was classified into Biological Process (BP), Cellular Component (CC) and Molecular Function (MF). There were 51 entries for BP, 4 for CC, and 9 for MF, respectively (Fig. **4A**). In BP mainly enriched miRNAs were involved in the positive regulation of ROS metabolism, cell cycle regulation, osteoclast differentiation, and chondrocyte differentiation; in CC, the cytoplasm, nucleus, and transcription factor complexes; and in MF, the protein phosphatase binding, sequence-specific DNA binding in the transcriptional regulatory region, and ubiquitin-protein ligase binding. In total, forty-five KEGG pathways were enriched, with the top 30 chosen to draw the bubble diagram (Fig. **4B**). MAPK signaling pathway, FoxO signaling pathway, GnRH signaling pathway and AGE-RAGE signaling pathway were mainly enriched in KEGG pathways.

### 3.4 Validation of Ferroptosis-associated Hub Gene Expression and its Diagnostic Value

**Fig. (5):**
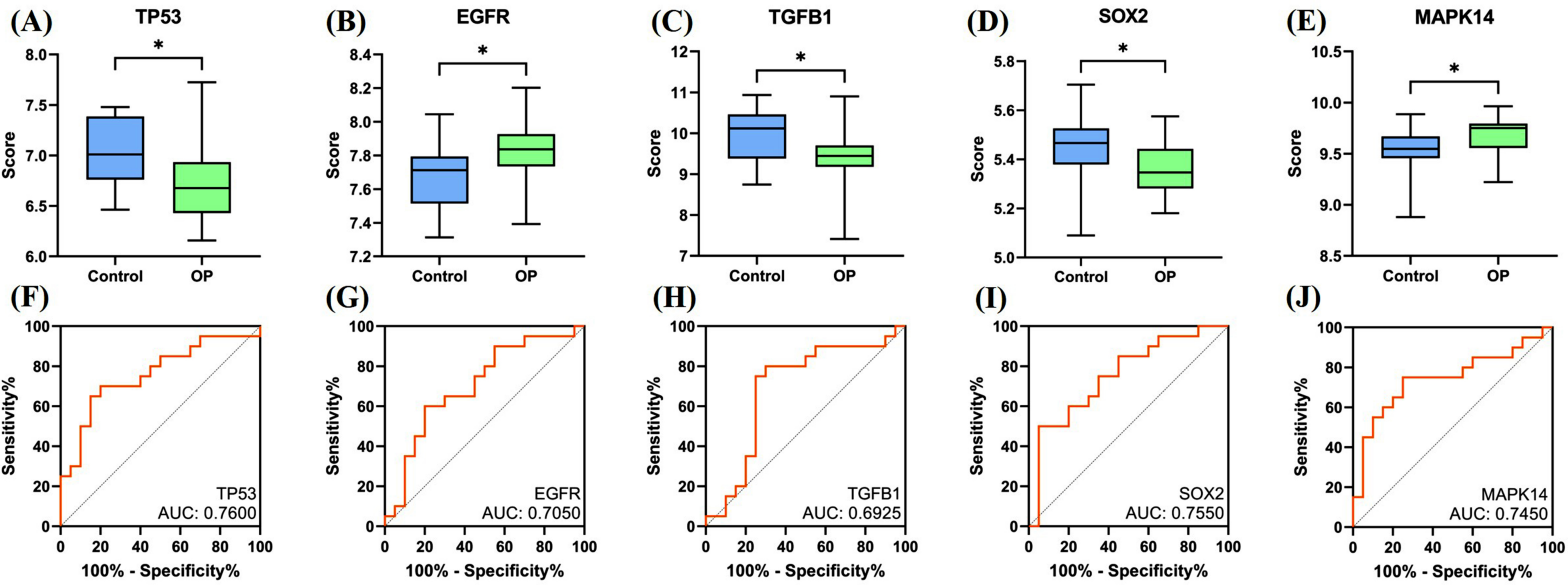
The expression and diagnostic value of the ferroptosis-related Hub genes. (**A**) The box plot of TP53 expression, (**B**) The box plot of EGFR expression, (**C**) The box plot of TGFB1 expression, (**D**) The box plot of SOX2 expression, (**E**) The box plot of MAPK14 expression, (**F**) ROC curve of TP53, (**G**) ROC curve of EGFR, (**H**) ROC curve of TGFB1, (**I**) ROC curve of SOX2, and (**J**) ROC curve of MAPK14.

Here, we added a validation step by introducing a new dataset from the GEO database: GSE56815, the results of which showed significant differences in the expression of five ferroptosis-related Hub genes such as TP53, EGFR, TGFB1, SOX2, MAPK14, between the osteoporosis group and normal controls (Figs. **5A-E**). We analyzed and plotted the receiver operating characteristic curve (ROC), quantifying the AUC, the genes with AUC > 0.6 were considered to contain diagnostic value. The AUC of TP53, EGFR, TGFB1, SOX2 and MAPK14 was found to be 0.7600, 0.7050, 0.6925, 0.7550, and 0.7450, respectively (Figs. **5F-J**), which could be indicated to be of significant value in the diagnosis of osteoporosis.

### 3.5 Network Construction of mRNA-miRNA

**Fig. (6):**
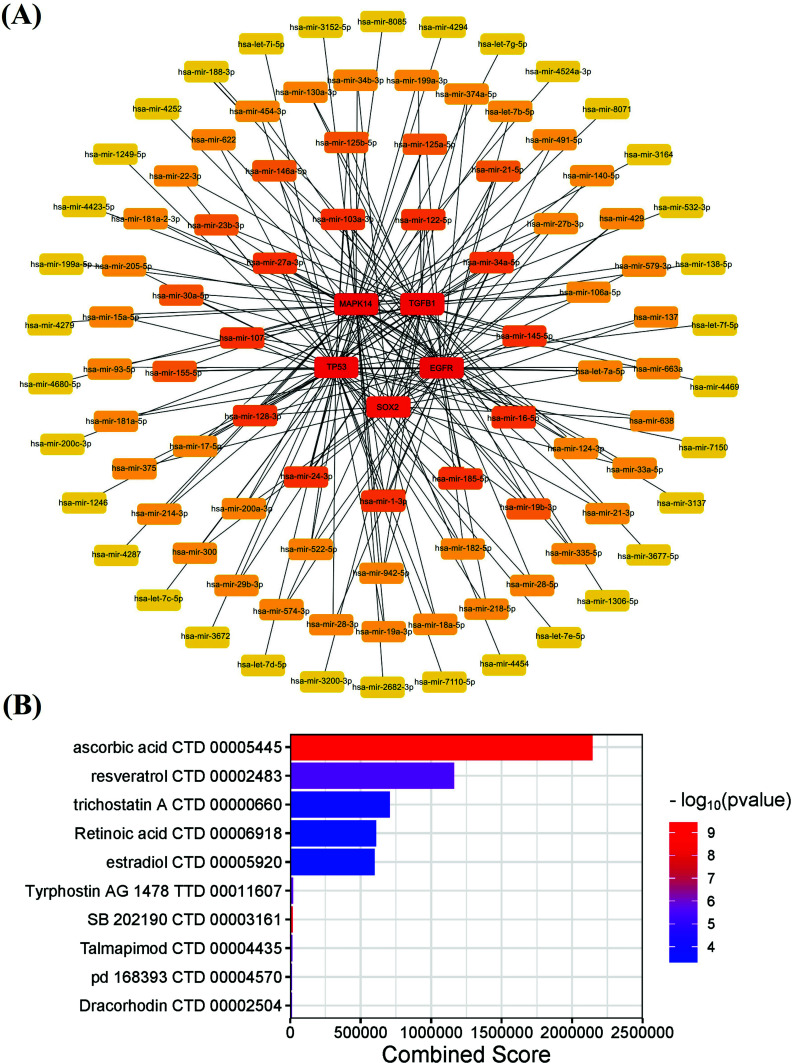
The network diagram of mRNA-miRNA and diagram of the targeted drug prediction. (**A**) The network diagram of mRNA-miRNA of five ferroptosis-related Hub genes, (**B**) The top 10 targeted drug prediction of five ferroptosis-related Hub genes.

We further investigated these five ferroptosis-related Hub genes, using the miRNet database to predict their upstream miRNAs, thanks to which, we obtained 359 target miRNAs and paired 461 mRNA-miRNAs. Of them, 174 miRNAs were found to regulate TP53; 83, to regulate EGFR; 46, to regulate TGFB1; 34, to regulate SOX2, and 124, to regulate MAPK14. When the predictive data were imported into Cytoscape (v3.9.1), the top 100 miRNAs with a binding degree were selected for the drawing of the co-expression network of mRNA and miRNA (Fig. **6A**).

### 3.6 Targeted Drug Prediction of Ferroptosis-related Hub Genes

Based on the DSigDB database, we identified Hub genes that were related to ferroptosis, predicting that these targeted drugs had the capacity of treating osteoporosis with ferroptosis regulated. A total of 1,232 drug molecules were predicted; the top 10 targeted drugs were selected and ranked by their combined scores, as indicated in the bar graph (Fig. **6B**). Actually, some of these compounds are well known in the field of osteoporosis, such as resveratrol, vitamin C, retinoic acid and estradiol.

### 3.7 Molecular Docking

**Fig. (7):**
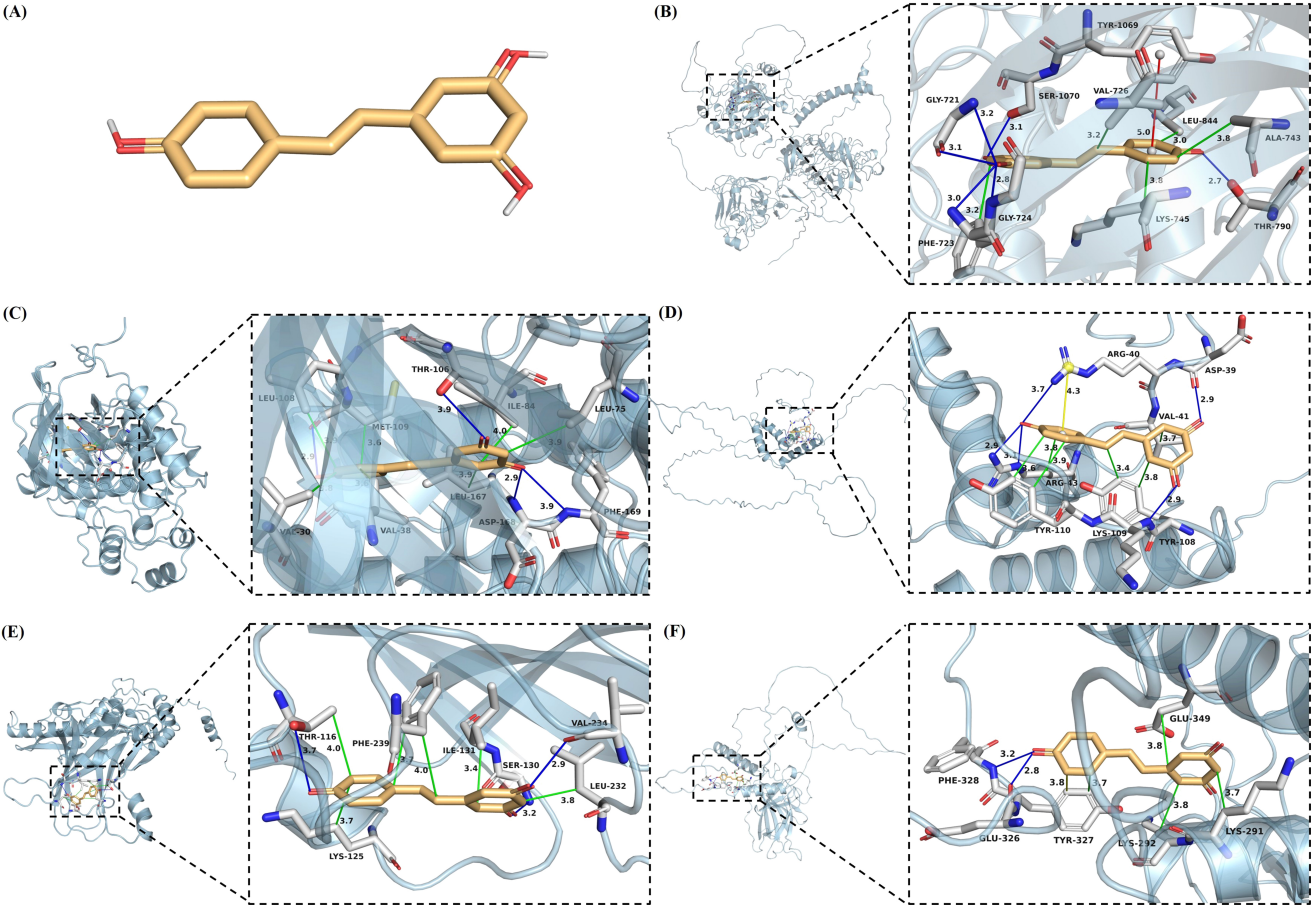
The molecular docking between resveratrol and five ferroptosis-related Hub proteins. (**A**) The structure of resveratrol, (**B**) The molecular docking between resveratrol and EGFR, (**C**) The molecular docking between resveratrol and MAPK14, (**D**) The molecular docking between resveratrol and SOX2, (**E**) The molecular docking between resveratrol and TGFB1, (**F**) The molecular docking between resveratrol and TP53. The white stick model representing the residues in the binding sites; the orange stick model, resveratrol; the green lines, the hydrophobic interactions; the blue lines, the hydrogen bonds; the red lines, π-stacking; the yellow lines, π-cation interactions; and the interaction distances indicated next to the bonds.

It is well-recognized that resveratrol plays an important role in both the alleviation of ferroptosis and the treatment of osteoporosis. In the drug prediction results, ascorbic acid was excluded because of its reactivity, and resveratrol had the second highest combined score. Therefore, we further explored the interaction between resveratrol and the five ferroptosis-associated hub genes, performing the molecular docking *via* AutoDock Vina (Fig. **7**).

As shown in Fig. (**7B**) on EGFR, resveratrol aroused a hydrophobic interaction with PHE723 (length = 3.25), VAL726 (length = 3.21), ALA743 (length = 3.79), LYS745 (length = 3.78) and LEU844 (length = 3.05); two hydrogen bonds with GLY721 (length = 3.18, 3.06), one hydrogen bond with PHE723 (length = 2.99), GLY724 (length = 2.80), THR790 (length = 2.70), SER1070 (length = 3.10), respectively; and a π-stacking with TYR1069 (length = 4.99).

As shown in Fig. (**7C**) on MAPK14, resveratrol developed a hydrophobic interaction with VAL30 (length = 3.76), VAL38 (length = 3.65), LEU75 (length = 3.86), ILE84 (length = 3.97), LEU108 (length = 3.87), MET109 (length = 3.56), LEU167 (length = 3.91); one hydrogen bond with THR106 (length = 3.95), MET109 (length = 2.94), ASP168 (length = 2.94) and PHE169 (length = 3.92), respectively.

As shown in Fig. (**7D**) on SOX2, resveratrol produced a hydrophobic interaction with VAL41 (length = 3.66), ARG43 (length = 3.84); two hydrophobic interactions with TYR108 (length = 3.44, 3.75), TYR110 (length = 3.61, 3.87); a hydrogen bond with ASP39 (length = 2.93), ARG40 (length = 3.66), LYS109 (length = 2.94); two hydrogen bonds with ARG43 (length = 3.11, 2.88), respectively; and a π-cation interaction with ARG40 (length = 4.30).

As shown in Fig. (**7E**) on TGFB1, resveratrol performed a hydrophobic interaction with THR116 (length = 3.98), LYS125 (length = 3.71), ILE131 (length = 3.41), LEU232 (length = 3.82); two hydrophobic interactions with PHE239 (length = 3.98, 3.72); a hydrogen bond with THR116 (length = 3.71), SER130 (length = 3.19), VAL234 (length = 2.91), respectively.

As shown in Fig. (**7F**) on TP53, resveratrol had a hydrophobic interaction with LYS291 (length = 3.71), LYS292 (length = 3.85), GLU349 (length = 3.85); two hydrophobic interactions with TYR327 (length = 3.76, 3.69); a hydrogen bond with GLU326 (length = 2.78), and PHE328 (length = 3.25), respectively.

In terms of binding affinity, the binding ability between the protein receptors and small molecular ligands was as follows: The lower the binding energy, the higher the binding affinity; the more stable the binding conformation of the protein receptor and small molecule ligand. The docking affinity showed that the active site of the protein target bound well with resveratrol, characterized by a strong degree (Table **1**).

## 4 DISCUSSION

In recent years, ferroptosis has received a growing amount of attention, for it is a newly discovered type of cell death. Iron is one of the most important trace elements in the human body, playing an important role in metabolism. Physiologically, the intestinal epithelial cells absorb ingested iron ions into the blood circulation and store them in the liver; the macrophages recover them from the red blood cells aging. In the body, iron balance is achieved by hepcidin in regulating iron transporters in tissues and serum [24]. The excess iron is then stored by ferritin in a non-toxic form [25]. Both iron deficiency and iron overload are known to cause a variety of diseases, including excessive ferrous ions promoting lipid peroxidation, producing lipid ROS, and inducing ferroptosis. Glutathione peroxidase 4 (GPX4) could maintain the redox balance of cells; therefore, regulating the GPX4 signaling pathway could regulate the ferroptosis of cells [26]. As indicated by the evidence that erastin could act as a ferroptosis inducer to inactivate GPX4 by depleting reduced glutathione and oxidized glutathione and that RSL3, another ferroptosis inducer, could directly and competitively bind to GPX4, resulting in the inactivation of GPX4 pathway; both could be capable of inhibiting GPX4, producing ROS, and promoting the occurrence of ferroptosis [27].

The problem is that osteoporosis is likely to be neglected, for its onset is so insidious that the condition is not detected until the occurrence of a fracture. In the elderly, however, osteoporotic fracture can be detrimental to the quality of life. In our study, we found that abnormal iron metabolism exerted a significant impact on osteoporosis. Osteoblasts are differentiated from mesenchymal stem cells in the bone marrow [28], which, when iron overload occurs, produce excessive ROS to activate the RIPK1-RIPK3-MLKL pathway, leading to ferroptosis of osteoblasts and eventually bone loss [29]. ROS activates the extracellular signal-regulated kinases and heat shock factor 2, stimulating RANKL expression [30]. When RANKL/OPG ratio is changed, the ratio of osteoblasts and osteoclasts is affected. Experiments have shown that iron overload increases intracellular ROS and the expression level of RANKL/OPG in cells, inhibiting the osteogenic ability of osteoblasts and stimulating osteoclast differentiation [31].

In our study, we screened out five ferroptosis-related osteoporosis Hub genes: TP53, EGFR, TGFB1, SOX2 and MAPK14 (Fig. **3B**). Of them, TP53, EGFR, TGFB1 and MAPK14 are the driver genes of ferroptosis, and SOX2 is the suppressor gene of ferroptosis [14]. Encoding p53 transcription factor, TP53 is the most frequently mutated tumor suppressor gene in all human cancer types, as manifested by the evidence that p53 could inhibit the expression of cystine and SLC7A11, a key component of glutamate antiporter, while reducing cellular antioxidant capacity and exacerbating ferroptosis [32]. By controlling the opening of the mitochondrial permeability transition pore, p53 caused osteoblast death in the patients on long-term glucocorticoids, leading to osteoporosis eventually [33]. In the human mammary epithelial cells, activated EGFR, through MAPK signaling, caused the cells to produce ROS, increasing catalase and NADPH oxidase 4 and promoting ferroptosis [34]. In osteoblasts, however, EGFR activated the MAPK/ERK pathway, stimulating the expression of downstream EGR transcription factor 2, which in turn-maintained cell growth, promoting cell proliferation and stimulating bone formation [35]. In the renal tubular cells cultured with high concentration of TGFB1, the concentration of glutathione was significantly decreased, leading to GPX4 inactivation and lipid peroxidation; the examination of these changes indicated that TGFB1 could induce ferroptosis, and these changes could be alleviated in the renal tubular cells treated with ferroptosis inhibitor, which indicated the ferroptosis effect of TGFB1 [36]. In hepatocellular carcinoma, TGFB1 caused redox imbalance, increasing the intracellular ROS level to promote ferroptosis [37]. TGFB1 was reported to be highly expressed in bone marrow mesenchymal stem cells (BMSCs) of type 2 diabetic rats; with TGFB1 inhibited, the osteogenic differentiation of BMSCs was enhanced, the aging of BMSCs delayed, the bone mass increased and the fracture healing promoted in the rats [38]. In the hypoxic cardiomyocytes, ROS levels were significantly reduced with MAPK14 inhibited, while they were significantly increased in those without MAPK14 inhibited [39]. Moreover, evidence showed that inhibition of MAPK14 blocked ferroptosis in testicular Sertoli cells during testicular ischemia-reperfusion [40]. In an ovariectomized rat model, oral administration of a MAPK14 inhibitor increased the levels of bone formation markers and reduced the loss of trabecular bone, which suggested that the MAPK14 pathway could regulate bone mass loss caused by estrogen deficiency [41]. In contrast to these genes, SOX2 plays a role in the repression of ferroptosis, as indicated by the evidence that SOX2 enhanced the resistance of lung cancer cells to ferroptosis by maintaining the level of SLC7A11 and regulating cysteine metabolism and GSH level in lung cancer cells [42]. In osteoblasts, SOX2 overexpression inhibited the transcription of miR-124-3p in rat BMSCs, and activated the PI3K/Akt/mTOR pathway, promoting the osteogenic differentiation of BMSCs [43]. Notably, these genes have all been reported to be related to ferroptosis or osteoporosis, and none of them have been found to be capable of regulating osteoporosis through ferroptosis. Therefore, further studies are needed to investigate the role of these genes in the regulation of ferroptosis in bone-related cells.

As indicated by our findings, many miRNAs could not only regulate the occurrence and development of osteoporosis, but also participate in the process of ferroptosis (Fig. **6A**), as indicated by the evidence that miR-27a-3p targeted SLC7A11 directly in non-small cell carcinoma cells to regulate ferroptosis [44]. In MC3T3-E1 cells, similarly, miR-27a-3p activated ERK1/2 signaling by targeting CRY2 to promote cell osteogenic differentiation [45].

From the subsequent pursuit of the relevant compounds which may play a potential targeting role in ferroptosis or osteoporosis, therefore, we succeeded in screening out top ten candidate chemicals (Fig. **6B**), of which resveratrol was found to be the most intriguing compound. Resveratrol is known to be a non-flavonoid polyphenolic organic compound with antioxidant, anti-inflammatory, and antimicrobial properties [46]. As demonstrated by the evidence, resveratrol performed such functions as enhancing the expression of GPX4, maintaining the level of SLC7A11, resisting cell ferroptosis and alleviating myocardial cell injury in the rats with myocardial injury [47]. Additionally, resveratrol has been reported to increase the osteogenesis of osteoblasts and increase BMD in the ovariectomized mice; the micro-CT scanning of the aged mice’s femurs indicated the BMD was significantly higher in the resveratrol-treated group than in the control group [48].

The examination of all these findings highlighted the evidence that resveratrol could have a promising potential for anti-ferroptosis capacity and osteoporosis treatment. Therefore, we continued to conduct in-depth research on resveratrol usingAlphaFold, an artificial intelligence tool, to successfully predict the protein structures of five target genes, and with the help of molecular docking technology, we came to know that resveratrol had a strong capacity for binding with five proteins (Fig. **7**). In the molecular docking model of EGFR and resveratrol, we discovered that the compound interacted with the five amino acid residues of GLY721, PHE723, GLY724, THR790 and SER1070 to generate six hydrogen bonds, thus generating a huge binding force (Fig. **7B**).

In MAPK14, moreover, resveratrol was found to be firmly encapsulated in the hydrophobic binding pocket, which was interactively formed with seven amino acid residues of VAL30, VAL38, LEU75, ILE84, LEU108, MET109 and LEU167 (Fig. **7C**), which could be explained by the evidence that the generation of a large number of hydrophobic interactions may change the spatial structure of the protein, triggering changes in protein function. This was also the case for the other four molecular docking models.

As indicated in Fig. (**7D**), additionally, the aromatic moiety on resveratrol was found to produce π-cation interaction with the protein charge center in the amino acid residue of ARG40 on SOX2. Under physiological conditions, the force of π-cation interaction is comparable to that of hydrogen bonding, as manifested in previously reported evidence that in the human brain, the π-cation interaction between nicotine and Trp residues in acetylcholine receptor could contribute to nicotine addiction [49]. Another force associated with the aromatic moiety, π-stacking occurred in the molecular model of resveratrol and EGFR, linked to the amino acid residue of TYR1069 (Fig. **7B**), which suggests that π-stacking itself is the interaction that occurs between aromatic rings. A recent study has shown a promising anti-tumor effect in a mouse model of oral tongue squamous cell carcinoma using the π-stacking method of assembling the photosensitizer chlorin with ferroptosis inducer erastin [50].

Resveratrol has the disadvantages of poor bioavailability and short half-life [51]. Therefore, molecular design of key functional groups is needed to enhance the biological activity of resveratrol. The molecular formula of resveratrol is C_14_H_12_O_3_, and its functional groups include a phenolic hydroxyl group, carbon-carbon double bond and aromatic ring. These functional groups link resveratrol to proteins. In our molecular docking results, these functional groups generated a total of 20 hydrogen bonds, 29 hydrophobic interactions, a π-cation interaction and a π-stacking with the five proteins. These functional groups offer great potential for modification of resveratrol. Pterostilbene was formed by replacing the hydroxyl group of resveratrol with the methoxy group. Compared with resveratrol, it has stronger lipophilicity and structural stability and has better pharmacokinetic properties [52, 53]. At the same time, many studies have added hydroxyl or halogen groups to the structure of resveratrol to form hydroxylated derivatives and halogenated derivatives. These derivatives are superior to conventional resveratrol in terms of physical and chemical properties, pharmacokinetics, and therapeutic effects [54-57]. The aforementioned findings helped us gain a deep insight into the mechanism of resveratrol binding itself with its targets, which we believe contributes to a better understanding of the pharmaceutical mechanism of action.

## CONCLUSION

In conclusion, we identified five ferroptosis-related osteoporosis Hub genes: TP53, EGFR, TGFB1, SOX2 and MAPK14. With the drug targeting prediction with molecular docking, we discovered resveratrol to highly target these five genes. Furthermore, we examined the relationship between ferroptosis and osteoporosis based on bioinformatics and network pharmacology, presenting a promising direction to the pursuit of the exact molecular mechanism of osteoporosis so that a new potential target can be discovered for the treatment of osteoporosis.

## AUTHORS’ CONTRIBUTIONS

Jin Shao and Weiwei Zhang designed the study. Kailun Huo, Yiqian Yang, Tieyi Yang, Weiwei Zhang, and Jin Shao were responsible for the experiments, data collection, literature review, and manuscript preparation. Kailun Huo performed the bioinformatics analysis. Jin Shao and Weiwei Zhang organized the structure of the manuscript, overseeing the language edit and revision and assisting in polishing the manuscript for its final publication. All authors read and approved the final manuscript.

## Figures and Tables

**Table 1:** The molecular docking binding energy of the resveratrol-bound hub proteins.

**Hub Proteins**	**ID in AlphaFold DB**	**Binding Affinity (kcal/mol)**
EGFR	AF-P00533-F1	-7.30
MAPK14	AF-Q16539-F1	-7.20
SOX2	AF-P48431-F1	-6.30
TGFB1	AF-P01137-F1	-6.90
TP53	AF-P04637-F1	-6.90

## Data Availability

The data and supportive information is available within the article.

## LIST OF ABBREVIATIONS

AUC: Area Under the ROC Curve
BMD: Bone Mineral Density
BP: Biological Process
CC: Cellular Component
GEO: Gene Expression Omnibus
GO: Gene Ontology
KEGG: Kyoto Encyclopedia of Genes and Genomes
MF: Molecular Function
PPI: Protein-protein Interaction
ROC: Receiver Operating Characteristic Curve

## References

[1] Compston J.E., McClung M.R., Leslie W.D. (2019). Osteoporosis.. Lancet.

[2] Brown C. (2017). Staying strong.. Nature.

[3] Lane N.E. (2006). Epidemiology, etiology, and diagnosis of osteoporosis.. Am. J. Obstet. Gynecol..

[4] Yang Y., Lin Y., Wang M., Yuan K., Wang Q., Mu P., Du J., Yu Z., Yang S., Huang K., Wang Y., Li H., Tang T. (2022). Targeting ferroptosis suppresses osteocyte glucolipotoxicity and alleviates diabetic osteoporosis.. Bone Res..

[5] Liu P., Wang W., Li Z., Li Y., Yu X., Tu J., Zhang Z. (2022). Ferroptosis: A new regulatory mechanism in osteoporosis.. Oxid. Med. Cell. Longev..

[6] Jiang X., Stockwell B.R., Conrad M. (2021). Ferroptosis: Mechanisms, biology and role in disease.. Nat. Rev. Mol. Cell Biol..

[7] Hadian K., Stockwell B.R. (2020). SnapShot: Ferroptosis.. Cell.

[8] Chen X., Kang R., Kroemer G., Tang D. (2021). Broadening horizons: The role of ferroptosis in cancer.. Nat. Rev. Clin. Oncol..

[9] Chen X., Kang R., Kroemer G., Tang D. (2021). Ferroptosis in infection, inflammation, and immunity.. J. Exp. Med..

[10] Li N., Jiang W., Wang W., Xiong R., Wu X., Geng Q. (2021). Ferroptosis and its emerging roles in cardiovascular diseases.. Pharmacol. Res..

[11] Xia Y, Zhang H, Wang H (2022). Identification and validation of ferroptosis key genes in bone mesenchymal stromal cells of primary osteoporosis based on bioinformatics analysis.. Front. Endocrinol..

[12] Luo C., Xu W., Tang X., Liu X., Cheng Y., Wu Y., Xie Z., Wu X., He X., Wang Q., Xiao Y., Qiu X., Tang Z., Shao G., Tu X. (2022). Canonical Wnt signaling works downstream of iron overload to prevent ferroptosis from damaging osteoblast differentiation.. Free Radic. Biol. Med..

[13] Barrett T., Wilhite S.E., Ledoux P., Evangelista C., Kim I.F., Tomashevsky M., Marshall K.A., Phillippy K.H., Sherman P.M., Holko M., Yefanov A., Lee H., Zhang N., Robertson C.L., Serova N., Davis S., Soboleva A. (2012). NCBI GEO: Archive for functional genomics data sets-update.. Nucleic Acids Res..

[14] Zhou N., Bao J. (2020). FerrDb: A manually curated resource for regulators and markers of ferroptosis and ferroptosis-disease associations.. Database..

[15] Szklarczyk D., Gable A.L., Nastou K.C., Lyon D., Kirsch R., Pyysalo S., Doncheva N.T., Legeay M., Fang T., Bork P., Jensen L.J., von Mering C. (2021). The STRING database in 2021: Customizable protein-protein networks, and functional characterization of user-uploaded gene/measurement sets.. Nucleic Acids Res..

[16] Shannon P., Markiel A., Ozier O., Baliga N.S., Wang J.T., Ramage D., Amin N., Schwikowski B., Ideker T. (2003). Cytoscape: A software environment for integrated models of biomolecular interaction networks.. Genome Res..

[17] Sherman B.T., Hao M., Qiu J., Jiao X., Baseler M.W., Lane H.C., Imamichi T., Chang W. (2022). DAVID: A web server for functional enrichment analysis and functional annotation of gene lists (2021 update).. Nucleic Acids Res..

[18] Chang L., Zhou G., Soufan O., Xia J. (2020). miRNet 2.0: Network-based visual analytics for miRNA functional analysis and systems biology.. Nucleic Acids Res..

[19] Yoo M., Shin J., Kim J., Ryall K.A., Lee K., Lee S., Jeon M., Kang J., Tan A.C. (2015). DSigDB: Drug signatures database for gene set analysis.. Bioinformatics.

[20] Kim S., Chen J., Cheng T., Gindulyte A., He J., He S., Li Q., Shoemaker B.A., Thiessen P.A., Yu B., Zaslavsky L., Zhang J., Bolton E.E. (2023). PubChem 2023 update.. Nucleic Acids Res..

[21] Jumper J., Evans R., Pritzel A., Green T., Figurnov M., Ronneberger O., Tunyasuvunakool K., Bates R., Žídek A., Potapenko A., Bridgland A., Meyer C., Kohl S.A.A., Ballard A.J., Cowie A., Romera-Paredes B., Nikolov S., Jain R., Adler J., Back T., Petersen S., Reiman D., Clancy E., Zielinski M., Steinegger M., Pacholska M., Berghammer T., Bodenstein S., Silver D., Vinyals O., Senior A.W., Kavukcuoglu K., Kohli P., Hassabis D. (2021). Highly accurate protein structure prediction with AlphaFold.. Nature.

[22] Forli S., Huey R., Pique M.E., Sanner M.F., Goodsell D.S., Olson A.J. (2016). Computational protein-ligand docking and virtual drug screening with the AutoDock suite.. Nat. Protoc..

[23] Eberhardt J., Santos-Martins D., Tillack A.F., Forli S. (2021). AutoDock vina 1.2.0: New docking methods, expanded force field, and python bindings.. J. Chem. Inf. Model..

[24] Zhao N., Zhang A.S., Enns C.A. (2013). Iron regulation by hepcidin.. J. Clin. Invest..

[25] Theil E.C. (2013). Ferritin: The protein nanocage and iron biomineral in health and in disease.. Inorg. Chem..

[26] Yang W.S., Stockwell B.R. (2016). Ferroptosis: Death by lipid peroxidation.. Trends Cell Biol..

[27] Yang W.S., SriRamaratnam R., Welsch M.E., Shimada K., Skouta R., Viswanathan V.S., Cheah J.H., Clemons P.A., Shamji A.F., Clish C.B., Brown L.M., Girotti A.W., Cornish V.W., Schreiber S.L., Stockwell B.R. (2014). Regulation of ferroptotic cancer cell death by GPX4.. Cell.

[28] Ponzetti M., Rucci N. (2021). Osteoblast differentiation and signaling: Established concepts and emerging topics.. Int. J. Mol. Sci..

[29] Tian Q., Qin B., Gu Y., Zhou L., Chen S., Zhang S., Zhang S., Han Q., Liu Y., Wu X. (2020). ROS-mediated necroptosis is involved in iron overload-induced osteoblastic cell death.. Oxid. Med. Cell. Longev..

[30] Bai X., Lu D., Liu A., Zhang Z., Li X., Zou Z., Zeng W., Cheng B., Luo S. (2005). Reactive oxygen species stimulates receptor activator of NF-kappaB ligand expression in osteoblast.. J. Biol. Chem..

[31] Ma J., Wang A., Zhang H., Liu B., Geng Y., Xu Y., Zuo G., Jia P. (2022). Iron overload induced osteocytes apoptosis and led to bone loss in Hepcidin^−/−^ mice through increasing sclerostin and RANKL/OPG.. Bone.

[32] Jiang L., Kon N., Li T., Wang S.J., Su T., Hibshoosh H., Baer R., Gu W. (2015). Ferroptosis as a p53-mediated activity during tumour suppression.. Nature.

[33] Zhen Y., Wang G., Zhu L., Tan S., Zhang F., Zhou X., Wang X. (2014). P53 dependent mitochondrial permeability transition pore opening is required for dexamethasone-induced death of osteoblasts.. J. Cell. Physiol..

[34] Poursaitidis I., Wang X., Crighton T., Labuschagne C., Mason D., Cramer S.L., Triplett K., Roy R., Pardo O.E., Seckl M.J., Rowlinson S.W., Stone E., Lamb R.F. (2017). Oncogene-selective sensitivity to synchronous cell death following modulation of the amino acid nutrient cystine.. Cell Rep..

[35] Chandra A., Lan S., Zhu J., Siclari V.A., Qin L. (2013). Epidermal growth factor receptor (EGFR) signaling promotes proliferation and survival in osteoprogenitors by increasing early growth response 2 (EGR2) expression.. J. Biol. Chem..

[36] Kim S., Kang S.W., Joo J., Han S.H., Shin H., Nam B.Y., Park J., Yoo T.H., Kim G., Lee P., Park J.T. (2021). Characterization of ferroptosis in kidney tubular cell death under diabetic conditions.. Cell Death Dis..

[37] Kim D.H., Kim W.D., Kim S.K., Moon D.H., Lee S.J. (2020). TGF-β1-mediated repression of SLC7A11 drives vulnerability to GPX4 inhibition in hepatocellular carcinoma cells.. Cell Death Dis..

[38] Zhang P., Zhang H., Lin J., Xiao T., Xu R., Fu Y., Zhang Y., Du Y., Cheng J., Jiang H. (2020). Insulin impedes osteogenesis of BMSCs by inhibiting autophagy and promoting premature senescence *via* the TGF-β1 pathway.. Aging..

[39] Ashraf M.I., Ebner M., Wallner C., Haller M., Khalid S., Schwelberger H., Koziel K., Enthammer M., Hermann M., Sickinger S., Soleiman A., Steger C., Vallant S., Sucher R., Brandacher G., Santer P., Dragun D., Troppmair J. (2014). A p38MAPK/MK2 signaling pathway leading to redox stress, cell death and ischemia/reperfusion injury.. Cell Commun. Signal..

[40] Li L., Hao Y., Zhao Y., Wang H., Zhao X., Jiang Y., Gao F. (2018). Ferroptosis is associated with oxygen-glucose deprivation/reoxygenation-induced Sertoli cell death.. Int. J. Mol. Med..

[41] Caverzasio J., Higgins L., Ammann P. (2008). Prevention of trabecular bone loss induced by estrogen deficiency by a selective p38alpha inhibitor.. J. Bone Miner. Res..

[42] Wang X., Chen Y., Wang X., Tian H., Wang Y., Jin J., Shan Z., Liu Y., Cai Z., Tong X., Luan Y., Tan X., Luan B., Ge X., Ji H., Jiang X., Wang P. (2021). Stem cell factor SOX2 confers ferroptosis resistance in lung cancer *via* upregulation of SLC7A11.. Cancer Res..

[43] Gan L., Leng Y., Min J., Luo X.M., Wang F., Zhao J. (2022). Kaempferol promotes the osteogenesis in rBMSCs *via* mediation of SOX2/miR-124-3p/PI3K/Akt/mTOR axis.. Eur. J. Pharmacol..

[44] Lu X., Kang N., Ling X., Pan M., Du W., Gao S. (2021). MiR-27a-3p promotes non-small cell lung cancer through SLC7A11-mediated-ferroptosis.. Front. Oncol..

[45] Ren L.R., Yao R.B., Wang S.Y., Gong X.D., Xu J.T., Yang K.S. (2021). MiR-27a-3p promotes the osteogenic differentiation by activating CRY2/ERK1/2 axis.. Mol. Med..

[46] Chen X., Song X., Zhao X., Zhang Y., Wang Y., Jia R., Zou Y., Li L., Yin Z. (2022). Insights into the anti-inflammatory and antiviral mechanisms of resveratrol.. Mediators Inflamm..

[47] Liu J., Zhang M., Qin C., Wang Z., Chen J., Wang R., Hu J., Zou Q., Niu X. (2022). Resveratrol attenuate myocardial injury by inhibiting ferroptosis *via* inducing KAT5/GPX4 in myocardial infarction.. Front. Pharmacol..

[48] Pearson K.J., Baur J.A., Lewis K.N., Peshkin L., Price N.L., Labinskyy N., Swindell W.R., Kamara D., Minor R.K., Perez E., Jamieson H.A., Zhang Y., Dunn S.R., Sharma K., Pleshko N., Woollett L.A., Csiszar A., Ikeno Y., Le Couteur D., Elliott P.J., Becker K.G., Navas P., Ingram D.K., Wolf N.S., Ungvari Z., Sinclair D.A., de Cabo R. (2008). Resveratrol delays age-related deterioration and mimics transcriptional aspects of dietary restriction without extending life span.. Cell Metab..

[49] Xiu X., Puskar N.L., Shanata J.A.P., Lester H.A., Dougherty D.A. (2009). Nicotine binding to brain receptors requires a strong cation-π interaction.. Nature.

[50] Zhu T., Shi L., Yu C., Dong Y., Qiu F., Shen L., Qian Q., Zhou G., Zhu X. (2019). Ferroptosis promotes photodynamic therapy: Supramolecular photosensitizer-inducer nanodrug for enhanced cancer treatment.. Theranostics.

[51] Das S., Lin H.S., Ho P.C., Ng K.Y. (2008). The impact of aqueous solubility and dose on the pharmacokinetic profiles of resveratrol.. Pharm. Res..

[52] Kosuru R., Rai U., Prakash S., Singh A., Singh S. (2016). Promising therapeutic potential of pterostilbene and its mechanistic insight based on preclinical evidence.. Eur. J. Pharmacol..

[53] Lin H.S., Yue B.D., Ho P.C. (2009). Determination of pterostilbene in rat plasma by a simple HPLC-UV method and its application in pre- clinical pharmacokinetic study.. Biomed. Chromatogr..

[54] Larrosa M., Barberán T.F.A., Espín J.C. (2004). The grape and wine polyphenol piceatannol is a potent inducer of apoptosis in human SK-Mel-28 melanoma cells.. Eur. J. Nutr..

[55] Chen W., Yeo S.C.M., Elhennawy M.G.A.A., Xiang X., Lin H.S. (2015). Determination of naturally occurring resveratrol analog trans-4,4′-dihydroxystilbene in rat plasma by liquid chromatography-tandem mass spectrometry: Application to a pharmacokinetic study.. Anal. Bioanal. Chem..

[56] Li X.Z., Wei X., Zhang C.J., Jin X.L., Tang J.J., Fan G.J., Zhou B. (2012). Hypohalous acid-mediated halogenation of resveratrol and its role in antioxidant and antimicrobial activities.. Food Chem..

[57] Lee E.J., Min H.Y., Joo Park H., Chung H.J., Kim S., Nam Han Y., Lee S.K. (2004). G2/M cell cycle arrest and induction of apoptosis by a stilbenoid, 3,4,5-trimethoxy-4′-bromo- cis-stilbene, in human lung cancer cells.. Life Sci..

